# Medication availability and economic barriers to adherence in asthma and COPD patients in low-resource settings

**DOI:** 10.1038/s41533-022-00281-z

**Published:** 2022-05-30

**Authors:** Aizhamal Tabyshova, Talant Sooronbaev, Azamat Akylbekov, Maamed Mademilov, Aida Isakova, Aidai Erkinbaeva, Kamila Magdieva, Niels H. Chavannes, Maarten J. Postma, Job F. M. van Boven

**Affiliations:** 1 University Medical Center Groningen, Groningen Research Institute for Asthma and COPD (GRIAC), University of Groningen, Groningen, The Netherlands; 2 Pulmonology Department, National Center of Cardiology and Internal Medicine named after M.M. Mirrakhimov, Bishkek, Kyrgyzstan; 3 Department of Health Sciences, Unit of Global Health, University of Groningen, University Medical Center Groningen, Groningen, The Netherlands; 4 Pulmonology Department, Jalal-Abad Regional Hospital, Jalal-Abad, Kyrgyzstan; 5 Kyrgyz State Medical Institute of Post-Graduating Training and Continuous Education, Bishkek, Kyrgyzstan; 6 Department of Public Health and Primary Care, Leiden University Medical Center, Leiden, The Netherlands; 7 Department of Economics, Econometrics & Finance, University of Groningen, Faculty of Economics & Business, Groningen, The Netherlands; 8 Department of Pharmacology & Therapy, Airlangga University, Surabaya, Indonesia; 9 Center of Excellence in Higher Education for Pharmaceutical Care Innovation, Padjadjaran University, Bandung, Indonesia; 10 Department of Clinical Pharmacy & Pharmacology, Medication Adherence Expertise Center of the northern Netherlands (MAECON), University Medical Center Groningen, University of Groningen, Groningen, The Netherlands

**Keywords:** Health care economics, Outcomes research, Chronic obstructive pulmonary disease, Asthma

## Abstract

Inhaled medication is essential to control asthma and COPD, but availability and proper adherence are challenges in low-middle income countries (LMIC). Data on medication availability and adherence in Central Asia are lacking. We aimed to investigate the availability of respiratory medication and the extent of financially driven non-adherence in patients with COPD and asthma in Kyrgyzstan. A cross-sectional study was conducted in two regions of Kyrgyzstan. Patients with a physician- and spirometry confirmed diagnosis of asthma and/or COPD were included. The main outcomes were (1) availability of respiratory medication in hospitals and pharmacies, assessed by a survey, and (2) medication adherence, assessed by the Test of Adherence to Inhalers (TAI). Logistic regression analyses were used to identify predictors for adherence. Of the 300 participants (COPD: 264; asthma: 36), 68.9% were buying respiratory medication out-of-pocket. Of all patients visiting the hospital, almost half reported medication not being available. In pharmacies, this was 8%. Poor adherence prevailed over intermediate and good adherence (80.7% vs. 12.0% and 7.3%, respectively). Deliberate and erratic non-adherence behavior patterns were the most frequent (89.7% and 88.0%), followed by an unconscious non-adherent behavioral pattern (31.3%). In total, 68.3% reported a financial reason as a barrier to proper adherence. Low BMI was the only factor significantly associated with good adherence. In this LMIC population, poor medication availability was common and 80% were poorly adherent. Erratic and deliberate non-adherent behaviors were the most common pattern and financial barriers play a role in over two-thirds of the population.

## Introduction

Worldwide, COPD and asthma are the most prevalent chronic respiratory diseases placing a significant clinical and economic burden on patients and societies^1^. Notably, the burden of chronic respiratory diseases seems disproportionally high in low- and middle income countries (LMIC)^2,3^. Importantly, the higher the control of asthma and COPD and the lower their exacerbation rate, the lower the level of health care expenditures on these diseases^4,5^.

To control asthma and COPD, effective inhaled respiratory medicines have been developed and are widely recommended in clinical guidelines^6,7^. These medicines may however not all be available in all global settings, especially in LMIC. The World Health Organization (WHO) has developed the Essential Medicines List (EML), a list of essential medicines to help countries choose medicines that improve health and lower costs^8^. A survey of 32 LMIC indicated that over 90% had national EMLs in place^9^. While national EMLs aim to support the availability of a minimum number of respiratory drugs, actual, real-world, availability in LMIC is often much lower. Indeed, in LMIC, the availability of medicines for asthma was only 30.1% and 43.1%, in private and public sectors respectively^10^. An earlier study also found that the availability of three cornerstone asthma drugs (beclomethasone, budesonide, and salbutamol) in 52 LMIC was poor across treatment centers and hospitals^11^. More recently, a Ugandan study demonstrated that the majority of asthma and COPD medicines were largely unavailable, especially in public hospitals, and were unaffordable in the private sector^12^.

In addition, even when some medication may be available in LMIC, financial barriers may hamper proper adherence to inhalers in asthma and COPD patients. A significant association was found between non-adherence and poor disease outcomes (e.g., exacerbations), and greater health-economic burden (e.g., direct and indirect health care costs)^13,14^. Previous studies have shown that non-adherence can occur due to a number of reasons with cost-related reasons being an important one in high resource settings, with medicines potentially being considered not cost-effective^15^. The extent to which economic barriers play a role in medication non-adherence in LMIC is unknown. Assessing adherence to asthma/COPD medication has long been difficult to perform in LMIC settings given the lack of validated measurement methods. Especially for LMIC in the Central Asian region, limited data on medication availability and adherence has been published. Recently, the “Test of Adherence to Inhalers” (TAI) for asthma and COPD patients was developed, which potentially could provide required insights, also in LMIC. The TAI is a validated questionnaire to identify non-adherence and to assess barriers related to the use of inhalers in asthma and COPD, including financial reasons^16^.

The aim of this study is to describe availability of respiratory drugs and the extent of financially driven non-adherence in patients with COPD and asthma in a LMIC, taking Central Asian country Kyrgyzstan as a case study.

## Methods

### Study design

This was a cross-sectional study that was conducted from June 2021 to July 2021. We used questionnaires in a representative sample of patients with asthma and COPD. Ethical approval was obtained from the ethical committee of the National Center of Cardiology and Internal Medicine (NCCIM) with protocol number 4. The study is reported according to the STROBE checklist for cross-sectional studies (Supplementary Information).

### Setting

The study was conducted in two regions of Kyrgyzstan. Kyrgyzstan is a land-locked, mountainous lower-middle income country in Central Asia with, according to the Worldbank, a gross domestic product per capita of $1178 in 2020. The regions were Jalal-Abad and Chui (Bishkek). In the city of Jalal-Abad, patients were recruited in the primary health care center and at the pulmonology department of the Jalal-Abad regional hospital, and in Bishkek patients were recruited from several randomly selected primary health care centers.

### Participants

We included patients with asthma and COPD fulfilling the following inclusion and exclusion criteria.

- Inclusion criteria:
  - Age ≥18 years
  - Born and live in Kyrgyzstan and speaking Russian and/or Kyrgyz
  - Physician confirmed diagnosis of asthma/COPD
  - COPD: FEV_1_/FVC ratio <0.7
  - Asthma: Post-bronchodilator increase in FEV_1_ > 12% or 200 ml from baseline
  - Patient consent to participate and willing to sign the consent form
- Exclusion criteria:
  - FEV_1_/FVC ratio >0.7 in COPD patients
  - No reversibility after bronchodilation test in asthma patients
  - Patients that have a disability in communication

### Data sources

For this study, multiple primary (i.e., a patient questionnaire) and secondary data (i.e., clinical record data from the branches of primary health care centers) sources were used. The questionnaire was developed by the research team, piloted among a small group, and optimized before large-scale data collection. The collecting process for the patient questionnaires is described below. As part of our Data Management Plan, only anonymized data were shared between study site collaborators and the research team, limiting data to the part that was kept securely taking the participant’s privacy into consideration.

### Data collecting process

Data collection was performed by the study site collaborators who were healthcare workers at the Respiratory Department of each hospital. To minimize bias in the selection of patients, every third diagnosed COPD/asthma patient from the pulmonologist registries was selected and contacted by phone by one of the study site collaborators to invite them (asking them to bring all the medication they use).

Firstly, study site collaborators introduced the meaning and target of the study to patients and then invited them to take part in the study.

Secondly, if patients agreed (and signed the informed consent that was written in the questionnaire, see Supplementary Information) the diagnosis was confirmed. In patients who did not have spirometry in their case-record, spirometry was performed for confirmation.

Afterwards, patients received the survey: patients did the survey by themselves with help from the study site collaborators if needed. In case of misunderstanding of any questions, collaborators helped to explain them.

Finally, when patients finished their survey and submitted it to the study site collaborators, collaborators checked it for detecting any missing answers or mistakes and asked them to correct it.

### Data to be collected

The Test of Adherence to Inhalers (TAI) (Supplementary Information) was used to identify problems with adherence to inhalation therapy. The TAI is a validated questionnaire consisting of 10 items (with a scale of 1–5) to be completed by the patient and two questions (with a scale of 1–2) to be completed by a healthcare professional. In this study, we used the formally translated Russian version of the TAI as available from www.taitest.com. The total score is 50 for the 10-item TAI and 54 for the 12-item TAI. The TAI distinguishes good adherence (TAI-10 = 50), intermediate adherence (TAI-10 = 46–49), and poor adherence (TAI-10 < 46). In addition, the type of non-adherence can be assessed including erratic (sum of TAI questions 1–5: <25), deliberate (sum of TAI questions 6–10: <25) and unconscious non-adherence (sum of TAI questions 11–12: <4). We had particular interest in TAI question 10 that focuses on financial barriers for non-adherence^16^.

Medication availability in hospitals, private clinics, and pharmacies as well as having received a straightforward technique training instruction on inhaler use was assessed by a patient survey that also included several baseline characteristics (Supplementary Information).

### Outcomes

The main study outcomes were (1) availability of respiratory medication and inhaler training in the hospital, community pharmacy, and private clinic, as measured by the survey, and (2) adherence to respiratory medication, as measured with TAI questionnaire. Of the two outcomes, the availability of medication is a health system barrier for which we expected little impact of patient-related co-variables. For the other outcome, i.e., medication adherence, we hypothesized that some patient co-variates may impact this. Therefore, several predefined demographic (e.g., age, sex, education, work status), clinical (e.g., disease severity, comorbidities), pharmaceutical (daily regimen), and socio-economic (e.g. monthly income, self-buying of medication) predictors for non-adherence were collected based on literature and the authors’ experience (Supplementary Information).

### Statistical methods and sample size

Descriptive statistics (mean, median, SD, minimum, maximum) and the Chi-square test were used for the comparison of categorical variables. Univariable and multivariable regression analyses were used to assess associations between predictors and the outcomes (i.e. proper medication adherence, defined as a 10-item TAI score >45). Anticipating to a degree of non-adherence of 50%^17^, a regression model with 15 potential predicting variables for non-adherence and the estimated requirement of 10 events of non-adherence per variable in logistic regression analysis^18^, we aimed for the inclusion of 300 patients. Variables with *p* < 0.25 in univariable regression analyses were included in multivariable regression analyses^19^. Statistical analysis was performed with Statistical Package for the Social Sciences (SPSS, Chicago, IL, USA) (version 26.0 for Windows). Statistical significance was set at *p* < 0.05.

### Reporting summary

Further information on research design is available in the Nature Research Reporting Summary linked to this article.

## Results

### Population characteristics

In total, the study included 300 participants, of which 264 patients with COPD and 36 patients with asthma. In Table 1, the sociodemographic and clinical characteristics of the study population are shown. The majority of the participants were male (57.0%), with a mean age of 58.5 (SD = 11.8) years, mostly with professional educational level (42.3%), active working status (42.0%), never smokers (52.0%) and using biomass for heating/cooking (48.3%). The mean duration of asthma/COPD was 9.8 (SD = 6.4) years. More than half of the participants had moderate COPD (60.6%), i.e., GOLD II. The most frequent comorbidities were cardiovascular diseases (48.0%) and allergic rhinitis (16.3%). Twenty percent used ≥3 co-medications. Short-acting muscarinic antagonists (SAMA), short-acting beta agonists (SABA), and inhaled corticosteroids/long-acting beta agonists (ICS/LABA) were the most frequently prescribed inhalers (57.2, 37.3, and 12.7%). Only 1.0% of patients used a long-acting muscarinic antagonist (LAMA), due to unavailability and unaffordability. Of note, 68.9% of the patients were buying respiratory medication from their own pocket. Most of them (38.8%) used respiratory medication four times per day.

**Table 1:** Sociodemographic and clinical characteristics of asthma and/or COPD participants (*n* = 300).

	All (*n* = 300)	COPD group (*n* = 264)	Asthma group (*n* = 36)
Age, y			
Mean (SD)	58.5 (11.8)	59.8 (10.8)	49.1 (14.5)
Median (minimum; maximum)	60 (19; 100)	61 (19; 100)	49 (21; 77)
Sex			
Male, No. (%)	171 (57.0)	157 (59.5)	14 (38.9)
Female, No. (%)	129 (43.0)	107 (40.5)	22 (61.1)
BMI, kg/m^2^			
Mean (SD)	28.2 (6.2)	28 (5.9)	29.4 (7.9)
Median (minimum; maximum)	27.7 (16.2; 93.3)	27.6 (18; 93.3)	29 (16.2; 59.3)
Monthly income, USD$			
Mean (SD)	128.9 (113.2)	127.2 (109.6)	142 (138.5)
Median (minimum; maximum)	98.5 (0; 944.3)	94.4 (0; 944.3)	118 (0; 802.6)
Missing value	6	5	1
Education			
Primary/secondary, No. (%)	84 (28.2)	73 (27.9)	11 (30.6)
Professional, No. (%)	127 (42.3)	110 (42.0)	17 (47.2)
University, No. (%)	87 (29.2)	79 (30.2)	8 (22.2)
Missing value	2	2	–
Working status			
Working, No. (%)	126 (42.0)	110 (41.7)	16 (44.4)
Unemployed, No. (%)	49 (16.3)	39 (14.8)	10 (27.8)
Retired, No. (%)	125 (41.7)	115 (43.6)	10 (27.8)
Having insurance, No. (%)	297 (99.0)	261 (98.9)	36 (100.0)
Missing value	3	3	–
Smoking status			
Current smoker, No. (%)	50 (16.7)	46 (17.4)	4 (11.1)
Ex-smoker, No. (%)	94 (31.3)	85 (32.2)	9 (25.0)
Never smoker, No. (%)	156 (52.0)	133 (50.4)	23 (63.9)
Biomass using for heating/cooking, No. (%)			
Yes	145 (48.3)	131 (49.6)	14 (38.9)
No	155 (51.7)	133 (50.4)	22 (61.1)
Disease duration, years			
Mean (SD)	9.8 (6.4)	9.7 (6.5)	10.6 (5.9)
Median (minimum; maximum)	10 (1; 40)	10 (1; 40)	10 (1; 20)
Pulmonary function tests			
FEV_1_, % predicted			
Mean (SD)	53.5 (14.9)	53.4 (15.4)	53.7 (10.9)
Median (minimum; maximum)	55 (18; 83)	55 (18; 83)	55 (34; 72)
FEV_1_/FVC ratio, %			
Mean (SD)	59 (11.0)	58.2 (8.5)	65.6 (21.3)
Median (minimum; maximum)	60 (0; 83.4)	60 (30.5; 83.4)	72 (0; 83)
Reversibility, %			
Mean (SD)	–	–	23.2 (10.0)
Median (minimum; maximum)	–	–	18.5 (9; 48)
COPD, GOLD grade, No. (%)			
GOLD 1	–	3 (1.0)	–
GOLD 2	–	160 (60.6)	–
GOLD 3	–	79 (29.9)	–
GOLD 4	–	22 (8.3)	–
ACO, No. (%)	11 (3.7)		–
Comorbidities			
Cardiovascular diseases, No. (%)	144 (48.0)	135 (51.1)	9 (25.0)
Allergic rhinitis, No. (%)	49 (16.3)	27 (10.2)	22 (61.1)
Bronchiectasis, No. (%)	16 (5.3)	15 (5.7)	1 (2.8)
Diabetes, No. (%)	30 (10.0)	26 (9.8)	4 (11.1)
Depression, No (%)	7 (2.3)	6 (2.3)	1 (2.8)
Co-medications			
0 co-medication, No (%)	160 (53.3)	142 (53.8)	18 (50.0)
1 co-medication, No (%)	35 (11.7)	23 (8.7)	12 (33.3)
2 co-medications, No (%)	44 (14.7)	42 (15.9)	2 (5.6)
≥3 co-medications, No (%)	61 (20.3)	57 (21.6)	4 (11.1)
Type of prescribed medication			
SABA	112 (37.3)	88 (33.3)	24 (66.7)
SAMA	171 (57.2)	167 (63.5)	4 (11.1)
SABA/SAMA	34 (11.3)	28 (10.6)	6 (16.7)
LAMA	3 (1.0)	3 (1.1)	0 (0.0)
LABA	20 (6.7)	18 (6.8)	2 (5.6)
ICS/LABA	38 (12.7)	31 (11.7)	7 (19.4)
ICS	34 (11.3)	19 (7.2)	15 (41.7)
Xanthines	19 (6.3)	14 (5.3)	5 (13.9)
Mucolytics	15 (5.0)	13 (4.9)	2 (5.6)
Prednisolone	16 (5.3)	10 (3.8)	6 (16.7)
Antibiotics	23 (7.7)	19 (7.2)	4 (11.1)
Number of times taking respiratory medication per day			
1 time, No (%)	11 (3.7)	8 (3.0)	3 (8.3)
2 times, No (%)	92 (30.8)	74 (28.1)	18 (50.0)
3 times, No (%)	56 (18.7)	56 (21.3)	–
4 times, No (%)	116 (38.8)	111 (42.2)	5 (13.9)
5 times, No (%)	24 (8.0)	14 (5.3)	10 (27.8)
Missing value	1	1	–
Buying respiratory medication, No (%)			
Myself	206 (68.9)	184 (70.0)	22 (61.1)
Partly covered by health insurance	84 (28.1)	72 (27.4)	12 (33.3)
Fully covered by health insurance	9 (3.0)	7 (2.7)	2 (5.6)
Missing value	1	1	–
Co-payment for the drugs, mean (SD)			
Formal, USD$	8.3 (8.2)	7.9 (8.0)	10.5 (9.0)
Informal, USD$	2.1 (4.8)	1.6 (3.5)	5.3 (9.5)

Current rate of USD$ to KGS = 84.72 som (July, 2021).

*SD* standard deviation, *ACO* asthma, COPD overlap, *FEV*_1_ forced expiratory volume in 1 s; *FVC* forced vital capacity, *SABA* short-acting ß2-agonists, *SAMA* short-acting muscarinic antagonists, *LAMA* long-acting muscarinic antagonists, *LABA l*ong-acting ß2-agonists, *ICS* inhaled corticosteroids.

### Availability of respiratory medication

Table 2 shows the availability of respiratory medication and inhaler technique education. Of all patients that visited the hospital, almost half reported that medication was not available. For the pharmacy, this was the case in 8%. Around a third of the respondents visited a private clinic, yet medication was mostly not available. There was no any difference in availability for respiratory drugs between Bishkek and Jalal-Abad patients. Almost all study participants were trained to use respiratory inhalers, either by a doctor (90%), a pharmacist (6.3%), a nurse (2.3%), and only 1.3% was never trained.

**Table 2:** Availability of respiratory medication and inhaler technique education.

Variable	All (*n* = 300)	COPD group (*n* = 264)	Asthma group (*n* = 36)
Availability in the hospital			
Yes	95 (31.7)	83 (31.4)	12 (33.3)
No	86 (28.7)	66 (25.0)	20 (55.6)
Did not visit this one	119 (39.7)	115 (43.6)	4 (11.1)
Availability in the private clinic			
Yes	33 (11.0)	26 (9.8)	7 (19.4)
No	79 (26.3)	62 (23.5)	17 (47.2)
Did not visit this one	188 (62.7)	176 (66.7)	12 (33.3)
Availability in the pharmacy			
Yes	265 (88.7)	234 (88.6)	31 (86.1)
No	24 (8)	19 (7.2)	5 (13.0)
Did not visit this one	11 (3.7)	11 (4.2)	–
Trained how to use inhalers			
By doctor	270 (90.0)	242 (91.7)	28 (77.8)
By nurse	7 (2.3)	5 (1.7)	2 (5.6)
By pharmacist	19 (6.3)	14 (5.3)	5 (13.9)
Never	4 (1.3)	3 (1.1)	1 (2.8)

Data as frequencies and percentages in parenthesis.

### Adherence to respiratory medication

Adherence to inhalers was evaluated with the TAI questionnaire^16^ (Table 3). When assessing the 10-item TAI level of adherence in the total population, it was found that poor adherence prevailed over intermediate and good adherence (80.7% vs. 12.0% and 7.3%), with similar patterns observed in the asthma and COPD groups. The 12-item TAI revealed that the deliberate and erratic non-adherence behavior patterns were the most frequent (89.7% and 88.0%), followed by the unconscious behavior pattern (31.3%).

**Table 3:** Adherence to inhalers, adherence level and non-adherence behavior patterns in asthma and/or COPD patients.

Variable	All (*n* = 300)	COPD group (*n* = 264)	Asthma group (*n* = 36)
Adherence level (10-item TAI)			
Good	22 (7.3)	22 (8.3)	0 (0.0)
Intermediate	36 (12.0)	28 (10.6)	8 (22.2)
Poor	242 (80.7)	214 (81.1)	28 (77.8)
Non-adherence behavior (12-item TAI)			
Erratic	264 (88.0)	231 (87.5)	33 (91.7)
Deliberate	269 (89.7)	235 (89.0)	34 (94.4)
Unconscious	94 (31.3)	80 (30.3)	14 (38.9)

Data as frequencies and percentages in parenthesis.

*TAI* test of adherence to inhalers.

**Fig. 1:**
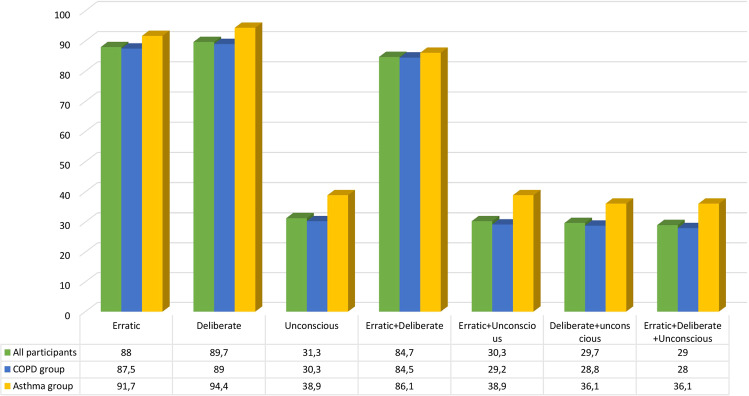
Percentages of non-adherence behavior patterns in asthma and COPD patients (12-item TAI).

As shown in Fig. 1, erratic behavior combined with deliberate behavior (84.7%) was more frequent than other combinations: erratic with unconscious (30.3%), deliberate with unconscious (29.7%), and erratic with deliberate and unconscious (29.0%), among which the difference was only a fraction of units.

**Fig. 2:**
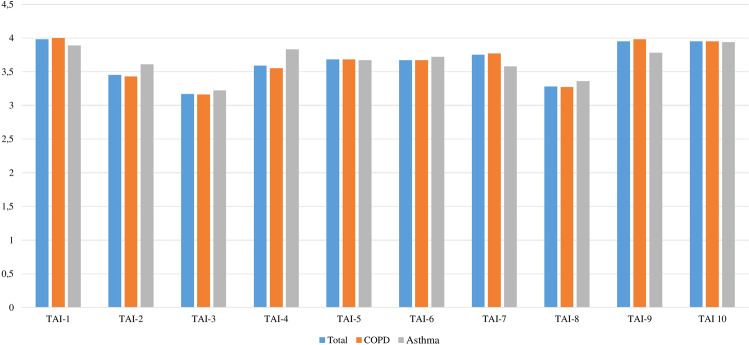
The mean scores of the individual barriers to medication adherence (10-item TAI). *TAI-1- the frequency of forgetfulness in the last 7 days; TAI-2 – forgetfulness generally; TAI-3 – stop taking inhalers when feeling well; TAI-4 – stop taking inhalers due to holidays; TAI-5 – sadness; TAI-6 –side effects; TAI-7 – belief of little help of inhalers; TAI-8 – deliberate taking fewer inhalations than prescribed by the doctor; TAI-9 – belief that inhalers interfere with day-to-day or work life; TAI-10 – finance troubles.

Of the individual barriers to medication adherence, stopping taking inhalers after the symptoms improve, deliberately reducing the number of inhalers prescribed by the doctor and forgetfulness were the TAI questions with the lowest scores (Fig. 2). Of all patients, 205 (68.3%) reported a financial reason (i.e., TAI question 10 score <5) to be a barrier to proper adherence.

### Associations with good adherence

The study population was distributed according to proper adherence (good or intermediate, defined as 10-item TAI > 45, i.e., *N* = 58) or non-adherence (poor, defined as 10-item TAI < 46, i.e., *N* = 242). In the multivariate regression analysis in the total population, having a low BMI was the only factor significantly associated with proper adherence (1.893, 95% CI 1.015–3.530). Male gender (1.235, 95% CI 0.656–2.327), low income (1.825, 95% CI 0.598–5.576), being unemployed (1.962, 95% CI 0.850–4.528), having allergic rhinitis (1.884, 95% CI 0.874–4.060), and previously trained in inhalation technique by a physician (6.868, 95% CI 0.895–52.691) were positively yet non-significantly associated with proper adherence (Table 4). Buying medication by the patient him or herself (0.641, 95% CI 0.340–1.208) and having to use medication more than two times per day (0.665, 95% CI 0.352–1.255) was negatively but non-significantly associated with proper adherence. Subgroup analyses in patients with either asthma or COPD showed similar patterns (Supplementary Information).

**Table 4:** Univariate and multivariate association with adherence in asthma and COPD patients (*n* = 300).

Variable	Univariate OR	*p*-value	Multivariate OR	*p*-value*
Age				
≤50 years	1.463 (95% CI 0.760–2.815)			
>50 years	Reference group	0.255		
Sex				
Male	1.420 (95% CI 0.785–2.568)	0.246	1.235 (95% CI 0.656–2.327)	0.513
Female	Reference group			
BMI				
Low BMI	1.777 (95% CI 0.988–3.198)	0.055	1.893 (95% CI 1.015–3.530)	0.045*
High BMI	Reference group			
Monthly income				
Low income	1.484 (95% CI 0.827–2.661)	0.186	1.825 (95% CI 0.598–5.576)	0.291
High income	Reference group			
Education				
Primary/secondary	1.044 (95% CI 0.484–2.252)	0.912		
Professional	1.088 (95% CI 0.542–2.183)	0.813		
University	Reference group			
Working status				
Working	0.887 (95% CI 0.462–1.701)	0.718		
Unemployed	1.774 (95% CI 0.824–3.821)	0.143	1.962 (95% CI 0.850–4.528)	0.114
Retired	Reference group			
Smoking status				
Current smoker	1.003 (95% CI 0.438–2.300)	0.993		
Ex-smoker	1.315 (95% CI 0.697–2.481)	0.398		
Never smoker	Reference group			
Biomass using for heating/cooking				
Yes	1.298 (95% CI 0.729–2.313)	0.376		
No	Reference group			
Disease duration				
>10 years	Reference group			
<10 years	1.415 (95% CI 0.779–2.571)	0.255		
Pulmonary function tests				
FEV_1_, % Predicted				
<52	Reference group			
>52	1.104 (95% CI 0.618–1.974)	0.738		
FEV_1_/FVC ratio, %				
<59	1.058 (95% CI 0.593–1.887)	0.850		
>59	Reference group			
COPD, GOLD grade				
GOLD 1–2	Reference group			
GOLD 3	1.081 (95% CI 0.551–2.122)	0.820		
GOLD 4	0.997 (95% CI 0.464–2.140)	0.993		
Comorbidities				
Cardiovascular diseases	0.854 (95% CI 0.480–1.518)	0.590		
Allergic rhinitis	2.134 (95% CI 1.070–4.257)	0.031*	1.884 (95% CI 0.874–4.060)	0.106
Diabetes	0.819 (95% CI 0.299–2.239)	0.697		
Buying respiratory medication				
Myself	0.568 (95% CI 0.314–1.028)	0.062	0.641 (95% CI 0.340–1.208)	0.169
Partly/fully covered by health insurance	Reference group			
Previous inhaler education				
By doctor	7.761 (95% CI 1.035–58.197)	0.046*	6.868 (95% CI 0.895–52.691)	0.064
By nurse/pharmacist/Never	Reference group			
Number of times taking medication per day				
1–2 times	Reference group			
>2 times	0.578 (95% CI 0.322–1.036)	0.066	0.665 (95% CI 0.352–1.255)	0.208

*BMI* body mass index, *FEV*_*1*_ forced expiratory volume in 1 s, *COPD* chronic obstructive pulmonary disease, *GOLD* The Global Initiative for Obstructive Lung Disease.

*p < 0.05; High BMI is >27.7 (median), Low BMI is <27.7 (median); High monthly income is >98.5 USD$ (median), Low monthly income is <98.5 USD$ (median). Missing variables: Monthly income –6; Education –2; Buying respiratory medication –1; Number of times taking medication per day –1.

## Discussion

In this study performed in a lower-middle income country, almost half of all asthma/COPD patients reported that medication was not available in the hospital. For the pharmacy, this was the case in 8%. Around 80% of the participants were poorly adherent. Erratic and deliberate non-adherent behavior were the most commonly observed patterns. Low BMI was the only factor significantly and positively associated with proper adherence in multivariable analyses. Male gender, low income, unemployed, having allergic rhinitis, and previously trained in inhalation technique by a physician were related with proper adherence but not significantly. Buying medication by the patient him or herself and having to use the medication more than two times per day was negatively but non-significantly associated with adherence. In addition, 68.3% of patients reported financial reasons as a barrier to good adherence. Regarding availability of respiratory medication, previously Zurdinova et al. reported that there were problems with the availability of inhaled medication in Kyrgyzstan. Inhaled corticosteroids (ICS) were only available for sale in Kyrgyz capital Bishkek and not in other regions. From the group of bronchodilators, only salbutamol and astalin were available all throughout Kyrgyzstan^20^. This seems reflected in our study with still the majority using short-acting medication (COPD: mostly short-acting muscarinic antagonists; asthma: mostly short-acting beta agonists) and only one third using long-acting medication (including ICS, which are now the first choice according to GINA). Importantly, of all COPD pharmacotherapy, only bronchodilators are economically affordable (in the range of the affordability coefficient from 0.1 to 0.4). ICS and fixed combinations of bronchodilators remain economically unaffordable (in the range of the affordability coefficient from 1.7 to 4)^20^. Indeed, previously we have shown that co-payments for COPD treatment in LMIC Kyrgyzstan can take up around 22% of patients’ annual income^21^. In the present study, there was no difference in the availability of respiratory medication between patients treated in Bishkek and Jalal-Abad. A positive finding was that for the patients who had medication available, almost all study participants were trained how to use respiratory inhalers, either by a doctor (90%), a pharmacist (6.3%), a nurse (2.3%), and only 1.3% was never trained. We found that “previously trained in inhalation technique by a physician” was positively yet non-significantly associated with adherence. This would particularly have an effect on the unconsciousness non-adherence type. Previous studies confirm that educational interventions on inhaler techniques are effective in stimulating proper medication use^22–24^.

Regarding the extent of adherence, our findings of 80% non-adherence are much poorer than reported for high-income countries that typically report rates of 50%^17^. However, exact rates of non-adherence also depend on the measurement method used and the underlying population. A study from Spain that also used the TAI to measure adherence in patients with asthma/COPD reported overall much higher good adherence rates of 49% for COPD and 28% for asthma^25^. This study also found erratic (asthma: 67%; COPD: 48%) and deliberate behavior (asthma: 47%; COPD: 34%) being the most frequent non-adherence behaviors, followed by unwitting behavior (asthma: 23%; COPD: 31%). This probably reflects the lack of educational programs and strategies to promote adherence.

Financial barriers to adherence have so far mostly been assessed in high-income settings. A study from Australia in patients with asthma reported high rates of cost-related underuse of asthma medicines (52.9% of adults and 34.3% of parents)^15^, yet in this LMIC study, these rates were almost double as high. This is hypothesized to be mostly attributed to higher rates of unaffordability. In turn, these economic barriers are associated with poor adherence. This issue can play an even more important role in this population where over 95% had to buy medication out-of-pocket or with co-payments given the lack of full health insurance coverage.

Our findings demonstrated that low BMI was the only factor significantly associated with adherence. This may be due to the fact that people with a lower BMI better monitor their health, paying also better attention to their nutrition and physical exercise. As such, they follow a healthier lifestyle including a better following of all the doctor’s prescriptions. This phenomenon has been previously observed in a large COPD trial and was described as the “healthy adherer effect”^26^. Also, it could be related to the fact that patients with low BMI have more advanced COPD and use therefore more medication.

The only predictors to non-adherence in the Spanish TAI study were “age under 50 years” and “active working status”^25^. In other studies, it was described that adherence was associated with age, current smoking status, number of respiratory drugs, number of daily respiratory drug doses and health-related quality of life^27–29^. In line with non-significant associations found in our study, males were more adherent than women, as found in a French study^30^, however, most studies have failed to identify any conclusive effect of gender on adherence^31–34^ and therefore we do not feel this issue requires attention. Lower socioeconomic status has been associated with poor adherence in asthma patients^33^, vis-a-vis in our study low income was non-significantly associated with adherence. Perhaps this was due to the fact that people with low incomes are observed in public clinics and are listed in the hospital register, while high-income patients usually go to private clinics or travel abroad for treatment, which has their own separate registers. It can also be explained by the fact that in Kyrgyz culture it is customary to help needy relatives financially, i.e., relatives buy inhalers and therefore patients strictly adhere to the medication regimen. Buying medication by the patient him or herself and having to use medication more than two times per day was negatively but non-significantly associated with good adherence. Perhaps, when a patient buys inhalers with his/her own money, he/she is more conscious of the value of the treatment and tries to follow the treatment regimen more strictly. The more frequent regimen being associated with poorer adherence is not surprising. In a previous review it was also was found that adherence was significantly higher for once-daily versus multiple dosing regimens^35^.

This study was the first investigating adherence in spirometry-confirmed COPD and asthma patients in a LMIC setting using the validated TAI questionnaire. The TAI questionnaire was not used in Kyrgyzstan before. The present research focused on barriers for adherence in LMIC, and was the first for Central Asia. Limitations include the fact that the number of patients with COPD and asthma was different and not comparable and therefore patients were analyzed together in the regression analyses. In addition, no disease control questionnaire was included while this may have been a predicting factor for adherence. Regarding the unwitting non-adherence aspect, the patients knew that their inhalation technique was evaluated, which could introduce bias. Also, the TAI questionnaire is not used in current daily Kyrgyz asthma/COPD management and they may therefore not be familiar with the type of questions.

Adherence to medication in COPD and asthma is important for clinical success, and non-adherence is a significant health and economic burden all over the world. Several simple methods can be applied to address this problem: using simplified medication regimens, increasing the patient’s knowledge of self-management, and improving the skills of health care workers in patient education and adherence counseling^36^. Smart inhalers have already been created, which are not yet used in practice, but have already yielded good results regarding monitoring and managing medication adherence, however, more research is needed^37^. It is important to note that there is a large shortage of primary health care workers, especially in more remote Kyrgyz regions, and due to the heavy workload of physicians, they physically do not have time or forget to evaluate or support patients’ adherence. Policy makers should focus on medication availability and affordability, increasing the primary health care workforce, and increasing awareness of patients on medication adherence. More research and interventions are needed to improve medication adherence in Kyrgyzstan and other LMIC.

While the availability of inhaled respiratory varied by facility, around 80% of patients in this LMIC population were poorly adherent. Erratic and deliberate non-adherent behaviors were the most common pattern and financial barriers play a role in 68.3% of the population.

## Supplementary information

Supplementary Information

REPORTING SUMMARY

## Data Availability

The datasets generated during and/or analyzed during the current study are available from the corresponding author on reasonable request.
