# Supplementary material for: Medication availability and economic barriers to adherence in asthma and COPD patients in low-resource settings
Source: NPJ Prim Care Respir Med. 2022 May 30;32:20. doi: 10.1038/s41533-022-00281-z (PMC9151780; doi:10.1038/s41533-022-00281-z)
Supplement: Supplementary file 1 — Supplementary Information [file 41533_2022_281_MOESM1_ESM.pdf]

Supplementary information

Medication availability and economic barriers to adherence in asthma and COPD patients in low-resource settings

STROBE checklist

STROBE Statement—Checklist of items that should be included in reports of ***cross-sectionalstudies***

	Item No	Recommendation	Page No
**Title and abstract**	1	(*a*) Indicate the study's design with a commonly used term in the title or the abstract	
		(*b*) Provide in the abstract an informative and balanced summary of what was done and what was found	
**Introduction**			
Background/rationale	2	Explain the scientific background and rationale for the investigation being reported	
Objectives	3	State specific objectives, including any prespecified hypotheses	
**Methods**			
Study design	4	Present key elements of study design early in the paper	
Setting	5	Describe the setting, locations, and relevant dates, including periods of recruitment, exposure, follow-up, and data collection	
Participants	6	(*a*) Give the eligibility criteria, and the sources and methods of selection of participants	
Variables	7	Clearly define all outcomes, exposures, predictors, potential confounders, and effect modifiers. Give diagnostic criteria, if applicable	
Data sources/ measurement	8*	For each variable of interest, give sources of data and details of methods of assessment (measurement). Describe comparability of assessment methods if there is more than one group	
Bias	9	Describe any efforts to address potential sources of bias	
Study size	10	Explain how the study size was arrived at	
Quantitative variables	11	Explain how quantitative variables were handled in the analyses. If applicable, describe which groupings were chosen and why	
Statistical methods	12	(*a*) Describe all statistical methods, including those used to control for confounding	

		(*b*) Describe any methods used to examine subgroups and interactions	
		(*c*) Explain how missing data were addressed	
		(*d*) If applicable, describe analytical methods taking account of sampling strategy	
		(*e*) Describe any sensitivity analyses	
**Results**			
Participants	13*	(a) Report numbers of individuals at each stage of study—eg numbers potentially eligible, examined for eligibility, confirmed eligible, included in the study, completing follow-up, and analysed	
		(b) Give reasons for non-participation at each stage	
		(c) Consider use of a flow diagram	
Descriptive data	14*	(a) Give characteristics of study participants (eg demographic, clinical, social) and information on exposures and potential confounders	
		(b) Indicate number of participants with missing data for each variable of interest	
Outcome data	15*	Report numbers of outcome events or summary measures	
Main results	16	(*a*) Give unadjusted estimates and, if applicable, confounder-adjusted estimates and their precision (eg, 95% confidence interval). Make clear which confounders were adjusted for and why they were included	
		(*b*) Report category boundaries when continuous variables were categorized	
		(*c*) If relevant, consider translating estimates of relative risk into absolute risk for a meaningful time period	
Other analyses	17	Report other analyses done—eg analyses of subgroups and interactions, and sensitivity analyses	
**Discussion**			
Key results	18	Summarise key results with reference to study objectives	
Limitations	19	Discuss limitations of the study, taking into account sources of potential bias or imprecision. Discuss both direction and magnitude of any potential bias	

Interpretation	20	Give a cautious overall interpretation of results considering objectives, limitations, multiplicity of analyses, results from similar studies, and other relevant evidence	
Generalisability	21	Discuss the generalisability (external validity) of the study results	
**Other information**			
Funding	22	Give the source of funding and the role of the funders for the present study and, if applicable, for the original study on which the present article is based	

*Give information separately for exposed and unexposed groups.

Note: An Explanation and Elaboration article discusses each checklist item and gives methodological background and published examples of transparent reporting. The STROBE checklist is best used in conjunction with this article (freely available on the Web sites of PLoS. Medicine at http://www.plosmedicine.org/, Annals of Internal Medicine at http://www.annals.org/, and Epidemiology at http://www.epidem.com/). Information on the STROBE Initiative is available at www.strobe-statement.org.

Informed consent

Your name: ____

Date:____

Agreement to participate in the study "Inhalation study"

The purpose of this activity is to obtain information about your disease and your adherence to taking an inhalation drug for the treatment of the disease.

We will use the information from this study to write a report. This report will be a public document. Your responses will be anonymous, which means that your names will not be used in any way in the report.

Your participation in this study is voluntary. You have the right to withdraw at any time during the study, for any reason. If you withdraw from the study, information collected from you, records and reports provided by you will not be used.

Please feel free to ask any questions about the study at any time. If you have any questions later, you can contact our research leader: Sooronbaev T.M., 0772574567. Your suggestions and concerns are very important to us.

I certify that I have been given information about participation in this study and that I understand it.

➔ **YES / NO** (please circle one of the two options)

And finally, we would like to ask your permission to photograph you during or after our joint work. If you agree with this, please circle 'yes'. If you don't want to be photographed, circle "no"

➔ **YES / NO** (please circle one of the two options)

Your first and last name____

Your signature____Date ________________

For the researcher:

I declare that I have informed the participant of my participation in this study.

Researcher Name: ____

Investigator Signature____________________________________Date_____________

Test of the Adherence to Inhalers (TAI) Questionnaire

1. How often did you forget to take your regular inhalers in the last 7 days?
☐ 1. Always ☐ 2. More than half ☐ 3. About half ☐ 4. Less than half ☐ 5. None

2. You forget to take your inhalers:
☐ 1. Always ☐ 2. Almost always ☐ 3. Sometimes ☐ 4. Almost never ☐ 5. Never

3. When you are feeling well, you stop taking your inhalers:
☐ 1. Always ☐ 2. Almost always ☐ 3. Sometimes ☐ 4. Almost never ☐ 5. Never

4. At the weekend or when you go on holiday, you stop taking your inhalers:
☐ 1. Always ☐ 2. Almost always ☐ 3. Sometimes ☐ 4. Almost never ☐ 5. Never

5. When you are anxious or sad, you stop taking your inhalers:
☐ 1. Always ☐ 2. Almost always ☐ 3. Sometimes ☐ 4. Almost never ☐ 5. Never

6. You stop taking your inhalers out of fear of potential side effects:
☐ 1. Always ☐ 2. Almost always ☐ 3. Sometimes ☐ 4. Almost never ☐ 5. Never

7. You stop taking your inhalers because you believe that they are of little help in treating your condition:
☐ 1. Always ☐ 2. Almost always ☐ 3. Sometimes ☐ 4. Almost never ☐ 5. Never

8. You take fewer inhalations than prescribed by your doctor:
☐ 1. Always ☐ 2. Almost always ☐ 3. Sometimes ☐ 4. Almost never ☐ 5. Never

9. You stop taking your inhalers because you believe that they interfere with your day-to-day or work life:
☐ 1. Always ☐ 2. Almost always ☐ 3. Sometimes ☐ 4. Almost never ☐ 5. Never

10. You stop taking your inhalers because you have trouble paying for them:
☐ 1. Always ☐ 2. Almost always ☐ 3. Sometimes ☐ 4. Almost never ☐ 5. Never

---

A healthcare professional responsible for the patient must answer the following two questions according to the data that appear the patient’s medical record (question 11) and after confirming their inhalation technique (question 12).

11. Does the patient know or remember the regimen (dose and frequency) that they were prescribed?
☐ 1. No ☐ 2. Yes

12. The patient's inhalation technique for the device:
☐ 1. Has critical errors ☐ 2. Has no critical errors or is correct

TOTAL SCORE

Patient questionnaire

Demographic	
Patient number	
What is your age?	years
Gender	(1) Male<br>(2) Female
Length	Cm
Weight	Kg
Do you have house mates?	(1) Yes, partner/spouse/friends<br>(2) Yes, children<br>(3) Yes, (grant)parents<br>(4) No, I live alone
What is your highest education?	(1) None<br>(2) Primary school<br>(3) Secondary school<br>(4) Professional<br>(5) University
Do you currently have a job?	(1) Yes<br>(2) No<br>(3) Retired
What kind of job?	
What is your monthly income?	
Do you have health insurance?	(1) Yes<br>(2) No
Risk factors	
Smoking status	(1) Current smoker<br>(2) Ex-smoker<br>(3) Never smoker
Do you use biomass for cooking and/or heating?	(1) Yes<br>(2) No
Clinical variables	
How many years have you been suffering from asthma/COPD?	
COPD:<br>• FEV1 (ml)<br>• FEV1/FVC ratio	
Asthma:<br>• FEV1 (ml)<br>• Reversibility % (ml)	
**Comorbidity**<br>• cardiovascular disease<br>• allergic rhinitis<br>• bronchiectasis<br>• diabetes<br>• depression/anxiety<br>• TB	Yes/no<br>Yes/no<br>Yes/no<br>Yes/no<br>Yes/no<br>Yes/no<br>Other diseases:

• Other, please specify	
Treatment	
-Did your doctor prescribe you any respiratory medication for your asthma and /or COPD?<br>-What medication have you been prescribed?<br>• SABA: (e.g. salbutamol, terbutaline)<br>• SAMA: (e.g. ipratropium)<br>• SABA/SAMA: (e.g. Berudual)<br>• LAMA: (e.g. Tiotropium, umeclidinium)<br>• LABA (e.g. formoterol, salmeterol, olodaterol, indacaterol)<br>• ICS/LABA: (e.g. seretide, symbicort)<br>• ICS: (e.g. beclomethasone, budesonide, fluticasone, mometasone)<br>• Xanthines: (e. g. theophylline, aminophylline)<br>• Mucolytics (e.g. acetulcystein, ambroxol)<br>• Prednisolone<br>• Antibiotics (specify which one)	Yes/No<br><br>Check which ones
• How many other medications do you have to use (not for asthma/COPD but for other disease, e.g. cardiovascular disease)?	
• How many times per day do you have to take your respiratory medication for your asthma/COPD?	(1) 1 time per day<br>(2) 2 times per day<br>(3) 3 times per day<br>(4) 4 times per day<br>(5) 5 or more times per day
• In the last year, were all your respiratory medications <u>always</u> available in the hospital?	(1) Yes<br>(2) No<br>(3) I did not visit this one
• Which ones were not available:	
• In the last year, were all your respiratory medications <u>always</u> available in the private clinic?	(1) Yes<br>(2) No<br>(3) I did not visit this one
• Which ones were not available:	
• In the last year, were all your respiratory medications <u>always</u> available in the pharmacy?	(1) Yes<br>(2) No<br>(3) I did not visit this one
• Which ones were not available:	
• Did you buy respiratory medication: (1) fully by yourself (2) partly covered by health insurance or (3) fully covered by the health insurance?<br>•	(1) Buy myself<br>(2) Partly covered by insurance<br>(3) Fully covered by insurance

• If not covered by insurance, how much do you have to pay yourself for drugs in addition? (formal payment)	
• If not covered by insurance, how much do you have to pay yourself for drugs in addition? (informal payment)	
• Have you been trained earlier how to use your inhalers?	(1) Yes, by doctor<br>(2) Yes, by nurse<br>(3) Yes, by pharmacist<br>(4) No, never

Comparison of adherence and its associations in asthma and COPD patients

When the study population was distributed according to proper adherence level (good or intermediate, defined as 10-item TAI>45, i.e. N=58) or non-adherence (poor, defined as 10-item TAI<46, i.e. N=242) to inhalers, in a bivariate comparison, there was an association between inhaler training by a doctor with adherence. Low BMI was related with adherence in the asthma group, yet numbers were small and no any association in the COPD group was observed (Supplementary Table 1).

Supplementary Table 1: Adherent versus non-adherent patients (total population, COPD and asthma groups)

	**All (n = 300)**			**COPD group (n = 264)**			**Asthma group (n = 36)**		
	**Adherent (n = 58)**	**Non-adherent (n = 242)**	**p - value**	**Adherent (n = 50)**	**Non-adherent (n = 214)**	**p - value**	**Adherent (n = 8)**	**Non-adherent (n = 28)**	**p - value**
Age			0.253			0.652			0.153
≤50 years	16 (24.2)	50 (75.8)		10 (21.3)	37 (78.7)		6 (31.6)	13 (68.4)	
>50 years	42 (17.9)	192 (82.1)		40 (18.4)	177 (81.6)		2 (11.8)	15 (88.2)	
Gender			0.245			0.296			0.465
Male	37 (21.6)	134 (78.4)		33 (21.0)	124 (79.0)		4 (28.6)	10 (71.4)	
Female	21 (16.3)	108 (83.7)		17 (15.9)	90 (84.1)		4 (18.2)	18 (81.8)	
BMI			0.053			0.182			0.049
Low BMI	36 (23.7)	116 (76.3)		30 (22.1)	106 (77.9)		6 (37.5)	10 (62.5)	
High BMI	22 (14.9)	126 (85.1)		20 (15.6)	108 (84.4)		2 (10.0)	18 (90.0)	
Monthly income			0.184			0.295			0.301
Low (<98.5$)	33 (22.4)	114 (77.6)		29 (21.8)	104 (78.2)		4 (28.6)	10 (71.4)	
High (>98.5$)	24 (16.3)	123 (83.7)		21 (16.7)	105 (83.3)		3 (14.3)	18 (85.7)	
Education			0.972			0.965			0.925
Primary/ Secondary	16 (19.0)	68 (81.0)		14 (19.2)	59 (80.8)		2 (18.2)	9 (81.8)	
Professional	25 (19.7)	102 (80.3)		21 (19.1)	89 (80.9)		4 (23.5)	13 (76.5)	
University	16 (18.4)	71 (81.6)		14 (17.7)	65 (82.3)		2 (25.0)	6 (75.0)	
Working status			0.190			0.260			0.783
Working	21 (16.7)	105 (83.3)		18 (16.4)	92 (83.6)		3 (18.8)	13 (81.3)	
Unemployed	14 (28.6)	35 (71.4)		11 (28.2)	28 (71.8)		3 (30.0)	7 (70.0)	
Retired	23 (18.4)	102 (81.6)		21 (18.3)	94 (81.7)		2 (20.0)	8 (80.0)	
Smoking status			0.672			0.621			0.990
Current smoker	9 (18.0)	41 (82.0)		8 (17.4)	38 (82.6)		1 (25.0)	3 (75.0)	
Ex-smoker	21 (22.3)	73 (77.7)		19 (22.4)	66 (77.6)		2 (22.2)	7 (77.8)	
Never	28 (17.9)	128 (82.1)		23 (17.3)	110 (82.7)		5 (21.7)	18 (78.3)	

smoker									
Biomass using for heating/ cooking			0.375			0.377			0.927
Yes	25 (17.2)	120 (82.8)		22 (16.8)	109 (83.2)		3 (21.4)	11 (78.6)	
No	33 (21.3)	122 (78.7)		28 (21.1)	105 (78.9)		5 (22.7)	17 (77.3)	
Disease						—			—
COPD	50 (18.9)	214 (81.1)	0.640	—	—	—	—	—	
Asthma	8 (22.2)	28 (77.8)	0.640	—	—	—	—	—	
ACO	1 (9.1)	10 (90.9)	0.381	—	—	—	—	—	
Pulmonary function tests									
FEV_1, % predicted			0.738			0.790			0.115
<52	24 (18.5)	106 (81.5)		23 (19.7)	94 (80.3)		01 (7.7)	12 (92.3)	
>52	34 (20.0)	136 (80.0)		27 (18.4)	120 (81.6)		7 (30.4)	16 (69.6)	
FEV_1/FVC ratio, %			0.850			0.688			0.720
<59	25 (19.8)	101 (80.2)		24 (20.0)	96 (80.0)		1 (16.7)	5 (83.3)	
>59	33 (19.0)	141 (81.0)		26 (18.1)	118 (81.9)		7 (23.3)	23 (76.7)	
Previous inhaler education			0.019			0.072			0.086
By doctor	57 (21.1)	213 (78.9)		49 (20.2)	193 (79.8)		8 (28.6)	20 (71.4)	
By nurse/ pharmacist	1 (3.3)	29 (96.7)		1 (4.5)	21 (95.5)		0	8 (100.0)	
Number of times taking medication per day			0.064			0.247			0.058
1 – 2 times	26 (25.2)	77 (74.8)		19 (23.2)	63 (76.8)		3 (33.3)	14 (66.7)	
>2 times	32 (16.3)	164 (83.7)		31 (17.1)	150 (82.9)		1 (6.7)	14 (93.3)	
Buying medication			0.060			0.172			0.120
Myself	34 (16.5)	172 (83.5)		31 (16.8)	153 (83.2)		3 (13.6)	19 (86.4)	
Partly/ Fully covered by health insurance	24 (25.8)	69 (74.2)		19 (24.1)	60 (75.9)		5 (35.7)	9 (64.3)	

* Bivariate analysis (Chi-square test) in the sociodemographic, clinical and functional variables according to adherence or non-adherence to inhalers in all sample studied and in COPD and asthma groups, ($p < .05$); High BMI is >27.7 (median), Low BMI is <27.7 (median); High monthly income is >98.5 USD$ (median), Low monthly income is <98.5 USD$ (median).
